# Optimal Tolerogenic Dendritic Cells in Type 1 Diabetes (T1D) Therapy: What Can We Learn From Non-obese Diabetic (NOD) Mouse Models?

**DOI:** 10.3389/fimmu.2019.00967

**Published:** 2019-05-14

**Authors:** David P. Funda, Lenka Palová-Jelínková, Jaroslav Goliáš, Zuzana Kroulíková, Alena Fajstová, Tomáš Hudcovic, Radek Špíšek

**Affiliations:** ^1^Institute of Microbiology of the Czech Academy of Sciences, v.v.i., Prague, Czechia; ^2^SOTIO a s., Prague, Czechia; ^3^Department of Immunology, 2nd Medical School, Charles University, Prague, Czechia

**Keywords:** type 1 diabetes, cell therapy, animal models, tolerogenic dendritic cells, NOD mouse, protocol optimization

## Abstract

Tolerogenic dendritic cells (tolDCs) are explored as a promising standalone or combination therapy in type 1 diabetes (T1D). The therapeutic application of tolDCs, including in human trials, has been tested also in other autoimmune diseases, however, T1D displays some unique features. In addition, unlike in several disease-induced animal models of autoimmune diseases, the prevalent animal model for T1D, the NOD mouse, develops diabetes spontaneously. This review compares evidence of various tolDCs approaches obtained from animal (mainly NOD) models of T1D with a focus on parameters of this cell-based therapy such as protocols of tolDC preparation, antigen-specific *vs*. unspecific approaches, doses of tolDCs and/or autoantigens, application schemes, application routes, the migration of tolDCs as well as their preventive, early pre-onset intervention or curative effects. This review also discusses perspectives of tolDC therapy and areas of preclinical research that are in need of better clarification in animal models in a quest for effective and optimal tolDC therapies of T1D in humans.

## Introduction

Type 1 diabetes (T1D) is a multifactorial, organ/cell-specific disease resulting from an autoimmune destruction of insulin-producing β cells of the endocrine pancreas by CD4^+^ and CD8^+^ T cells, as well as macrophages infiltrating the islets. The insulin deficiency together with suboptimal insulin replacement result in a complex metabolic derangement with abnormal metabolome (1, 2). The number of children and adolescents with T1D is estimated at 1,106,500 worldwide. The incidence of T1D is increasing more rapidly than expected and is causing a significant health problems and economic burden, also due to severe complications (e.g., diabetic retinopathy, neuropathy, kidney failure), (3). At present, no effective cure or secondary prevention of T1D exists. Although heavy immunosuppression, or a reset of the immune system by immunoablative therapy followed by autologous or allogeneic bone marrow transplantation, were able to stop/prevent recurrence of β-cell destruction, they have not been considered acceptable as treatments for T1D (4–7). Nevertheless, with recent advances, non-ablative autologous hematopoietic stem cell transplantation may yet come into its renaissance as a cure of T1D [reviewed (8)]. Apart from the above mentioned cases, no clinical trial has so far been able to establish remission of T1D in patients.

Dendritic cells (DCs) are specialized, potent antigen-presenting cells (APCs) that represent key regulators of immune responses, both innate and adaptive, effector and tolerance (9). Dendritic cells were first discovered by Steinman et al. in 1973, who described their immunostimulatory effect on T cells (10, 11). Under physiological conditions, when antigen presentation occurs without additional “danger” stimuli, DCs displaying immature character, steadily migrate to lymph nodes (LNs) and maintain peripheral tolerance in various tissue-specific environments (12, 13). Dendritic cells induce peripheral tolerance by various mechanisms including T-cell deletion, T-cell anergy and hyporesponsiveness, and the expansion of natural Tregs, inducible Tregs (14, 15), and Bregs (16). Although several different human and mouse DC subsets have been identified, and functionally specialized subsets exist [reviewed (17, 18)], it seems that their tolerogenic functions are not linked to a specific lineage or tissue subset and several micro-environmental factors (e.g., microbiom, apoptosis) may contribute to maintaining their tolerogenic character (19, 20).

Cell-therapies comprising tolerogenic DCs (tolDCs), Tregs or bone marrow transplantations represent novel emerging strategies for the treatment of autoimmune diseases (8, 21, 22). They also hold promise for the treatment of allergies (23–25), and may also improve transplantations (26). Both tolDCs and Tregs as *ex vivo* cell-therapies share certain disadvantages. For example, a requirement for extensive manipulations *in vitro* or their patient-specific, tailor-made character, makes their preparation laborious and expensive. Tolerogenic DCs display some specific advantages compared to Tregs. First, they act as central regulators of immune responses and may thus target Tregs at various check points [reviewed 17] lacking the clonality issues of T-cells (27), they possess good potential to migrate to immune inductive sites [reviewed (28, 29)]. Second, DCs are relatively easier to differentiate and to expand from peripheral blood monocytes separated by leukapheresis (30). Tolerogenic DCs are being tested in clinical trials as a potential cell-therapy for autoimmune diseases such as rheumatoid arthritis, multiple sclerosis, T1D and Crohn‘s disease [reviewed (31)]. At this moment, there is one completed and one ongoing phase I clinical trial with autologous tolDCs in patients with type 1 diabetes (32–34).

For T1D, the beginning of dendritic cell-based therapies goes back to the study by Clare-Salzler et al. who documented that DCs isolated from pancreatic lymph node (PLN), but not T cells or DCs from other lymph nodes, of 8-20-week-old NOD females prevented diabetes in 4-week-old recipient NOD mice (35). This study still poses questions requiring follow-up experimentation e.g., does the age of DC donors alter their disease-preventive effects, or do environmental factors influencing the penetrance of T1D modify the disease-preventive capacity of PLN DCs. Nevertheless, and more importantly, this study paved the road to DC-based cell therapies in T1D. Since then, several protocols of tolerogenic DCs have been developed, many using tolDCs without *in vitro* supplied antigen (36–42), although antigen-loaded tolDCs protocols have also been tested (42–46). These protocols have been applied to animal models of T1D, preferentially the non-obese diabetic (NOD) mice (21).

Animal models represent an irreplaceable tool in preclinical tolDCs testing. Many studies tolDCs studies have been carried out in the NOD mouse model, as it represents a very close (genetically, immunologically, and environmentally) and spontaneous model of the human disease, allowing one to study therapeutical interventions in the context of the natural history of type 1 diabetes (47, 48). NOD mice however display also several suboptimal features, among them defects in maturation of the myeloid lineage and myeloid DCs are indeed the most related to tolDCs testing (49, 50). Several other mouse models have been employed, albeit less frequently, such as the NOD-SCID model of adoptive cotransfer of diabetes (51), the NOD RIP-IFN-β mouse (44), the LCMV-RIP induced model (52), or humanized HLA-DQ8/RIP-B7.1 or HLA-DR4 mice (39, 46).

Because a wide array of protocols for tolDCs exists, preclinical testing of multiple parameters is both difficult and necessary. Various parameters of safe and effective tolDCs for T1D should be optimized in *in vitro* and in animal models (e.g., tolDC stability, homogeneity, survival, migration capacities). In addition to optimal antigen form and dose in case of antigen-loaded tolDCs, an optimal combination of cell dose, application scheme and application route should be determined. Only a few tolDC protocols, e.g., IL-4 transduced tolDCs, were able to cure or revert diabetes in NOD mice, thus other protocols should be tested in more animal models and attempts should be made not only to prevent, but also to stop the diabetogenic process before disease onset and/or to cure already diabetic animals (53, 54).

Compared to e.g., mucosal delivery of autoantigens as prevention/therapy of T1D (55, 56), using a cell entity for *in vivo* therapeutic effects represents a much more challenging scenario that requires thorough preclinical testing. Efforts have been made to standardize information provided for various protocols, models and data from preclinical testing of tolDCs in autoimmune diseases (57, 58).

While the therapeutic use of *in vivo* targeted tDCs via DEC-205 (9) or the use of plasmacytoid DCs (59) in T1D have already been reviewed, this review deals with animal testing of tolDCs prepared *in vitro* from mouse bone marrow precursors, that are almost exclusively used as a mouse parallel to human monocyte-derived tolDCs from peripheral blood mononuclear cells (PBMCs) (30). In this review we discuss the parameters of *in vitro* generated tolDCs in mouse models of T1D, the importance of protocol optimizations and what aspects are desirable to be further addressed in preclinical testing in animal models of T1D.

## Culture Conditions of tolDCs in T1D

### TolDCs *in vitro* Propagation

Most of the protocols applied in T1D use propagation of tolDCs from bone marrow progenitors in the presence of granulocyte-macrophage colony-stimulating factor (GM-CSF) and IL-4 (36, 37, 42, 45, 46, 53, 54, 60–66), while two groups reported GM-CSF and IL-10 (39, 43, 52). A few studies employed GM-CSF alone for *in vitro* generation of diabetes-preventive tolDCs (38, 44, 67). An overview of tolDCs protocols is provided in Table 1. Adoptively transferred *in vivo* generated GM-CSF DCs (69) decreased diabetes incidence in NOD mice as well. There are also reports supporting the role of TGF-β in DC-mediated disease protection. Thus, in another study targeting DCs *in vivo, s.c*. microparticle-encapsulated TGF-β was used to enhance diabetes protection of NOD mice (70) and *in vitro* GM-CSF generated bone marrow-derived DCs (BMDCs), conditioned for 24 h with TGF-β, prolonged islet graft survival in diabetic mouse recipients (71).

**Table 1:** Example protocols of tolDCs in T1D and extent of their preclinical testing.

**tolDC propagation**	**Modification**	**Culture condition**	**Stabilization**	**Unloaded/Ag_**1**_/Ag_**2**_**	**Ag dose**	**Cell dose**	**App. route and scheme**	**Model**	**Prevention**	**Pre-diabetic**	**Cure**	**References**
GM-CSF+IL-4	No	FCS	No	Yes/insulin B9-23 /GAD65_78−97_ /GAD65_260−279_	3 μM	1 × 10^5^	S.c. (footpad) 3 times, weekly or 3 times, weekly + every other week	NOD	Yes	Yes	–	(65)
GM-CSF+IL-4	Vitamin D2/Dex on day 6	FBS/serum-free	MPLA	Yes/GAD65/OVA/GAD65 peptide no. 35	1 μg or 2μg/ml	3 × 10^6^	I.p.	NOD	Yes	–	–	(42)
								NOD-SCID				
GM-CSF+IL-4	Microspheres with antisense oligos. CD40/CD80/CD86	FBS	No	Yes/insulin B9-23	5 μg	2 × 10^6^	S.c. 8 times, weekly	NOD	–	–	Yes	(64)
GM-CSF+IL-4	Antisense oligos. CD40/CD80/CD86	FBS	LPS	Yes/no	n.a.	2 × 10^6^	S.c. (abdominal) single or 8 times, weekly	NOD	–	–	Yes	(63)
GM-CSF+IL-4	No	FBS/serum-free	No	No/GAD65_217−236_	10 μg/ml	1 × 10^5^	I.v. 5 times, weekly	NOD	Yes	–	–	(66)
GM-CSF	No	FBS	No	Yes/NIT-1 apoptotic bodies	3 × 10^5^ cells	1 × 10^6^	I.p.	NOD	–	–	No	(68)
GM-CSF	No	FCS	No	Yes/no	n.a.	1 × 10^6^	I.p. i.v. 3 times, weekly	NOD-DQ8 RIP- B7.1	Yes	–	–	(39)
GM-CSF+IL-4												
GM-GSF+IL-10						3 × 10^6^	I.v.	NOD-SCID				
GM-CSF+IL-4	IL-4 transduced DCs (electroporated)	FCS	No	Yes/no	n.a.	1 × 10^6^	I.v.	NOD	–	Yes	Yes	(53)
GM-CSF+IL-10	No	FBS/normal mouse serum	No	Yes/2 peptides (insulin B9-23+insulin B15-23)	10 μg/ml	1 × 10^6^	I.p.	NOD	Yes	–	–	(43)
GM-CSF	No	FBS	No	Yes/NIT-1 or SV-T2 apoptotic bodies	3 × 10^5^ cells	1 × 10^6^	I.p.	NOD RIP-IFN.β	Yes	–	–	(44)
GM-CSF+IL-4	No	FCS	No	Yes/insulin B9-23 /proinsulin C19-A3/GAD65_78−97_	3 μM	1 × 10^5^	S.c. (footpad) 3 times, weekly	NOD	Yes	–	–	(45)
GM-CSF+IL-4	Antisense oligos. CD40/CD80/CD86	FBS	LPS	Yes/NIT-1 lyzate	n.a.	2 × 10^6^	I.p.	NOD	Yes	–	–	(36)
GM-CSF+IL-4	IL-4 transduced DCs (adenoviral vector)	FBS	No	Yes/no	n.a.	4–5 × 10^5^	I.v. single or 2 times, weekly	NOD	Yes	Yes	–	(54)
GM-CSF or GM-CSF+IL-4	No	FBS	No	Yes/3 peptides (hsp60_437−460_ +GAD65_509−528_ +GAD65_524−543_)	3 × 60 μg/ml	4–8 × 10^5^	I.v. 3 times, weekly	NOD	Yes	–	–	(37)

Although both GM-CSF+IL-4 propagated tolDCs, and to a lesser extent also GM-CSF tolDCSs, were shown to prevent diabetes, comparison of unloaded GM-CSF/IL-4 *vs*. GM-CSF/IL-10 *vs*. GM-CSF alone-cultured tolDCs carried out by Tai et al. documented diabetes-preventive effect only for GM-CSF+IL-10 cultured tolDCs (39). Two other studies testing IL-10 showed that the GM-CSF+IL-10 protocol is effective only for antigen-loaded tolDCs cultured in autologous serum (43, 52). GM-CSF tolDCs were inferior to GM-CSF+IL-4 generated ones in diabetes prevention in NOD mice, especially when cultured without antigen (37, 72). The importance of IL-4 in the propagation of effective tolDCs was further documented by several studies (54, 73, 74), including gene array analyses, mapping increased expression of co-stimulatory molecules and differences in cytokine/chemokine signatures (75). BMDCs cultured in GM-CSF showed suboptimal characteristics compared to cells generated in combination with IL-4, especially in serum-free conditions (73) that are more relevant for human DC-preparations. Markedly enhanced trafficking and functional capacities were reported for IL-4 and GM-CSF propagation of DCs (76). These data may thus explain the less satisfactory results obtained with GM-CSF prepared tolDCs. Later a more worrying message on the heterogeneity of bone marrow derived DCs appeared (77).

### Homogeneity of tolDCs

Helft et al. provided detailed and comprehensive data documenting that mouse BMDCs prepared by culture with GM-CSF consist of a heterogeneous cell population comprising both immature DCs but also monocyte-derived macrophages that are found within the CD11c^+^MHC-II^+^ cells (77). While a similar study on GM-CSF+IL-4 or GM-CSF+IL-10 propagated cells using gene expression profiling is not available, one can perhaps speculate that an addition of IL-4 or IL-10 is unlikely to fully overcome this problem. Not only bone marrow lymphoid precursors, but also early progenitors of mouse conventional and plasmacytoid DCs, express Flt3 (78). Monocytes, macrophages, osteoclasts and DCs share a common progenitor (MODP). Compared to human lineage commitment, a monocyte/macrophage and osteoclast bipotent progenitor (MDP) is described in the mouse, but a dedicated DC progenitor is currently not clearly identified (79). Apart from these inherent homogeneity issues at the level of bone marrow progenitors used for generation of tolDCs, contaminating cells (T cells and/or B cells and MHC II^+^ cells) were depleted by complement/Abs in some protocols (37, 43, 52, 54) or by MACS depletion using e.g., anti CD3, B220, and Gr-1 mAbs (40). Several other tolDCs protocols however did not employ this purification step.

**Figure 1:**
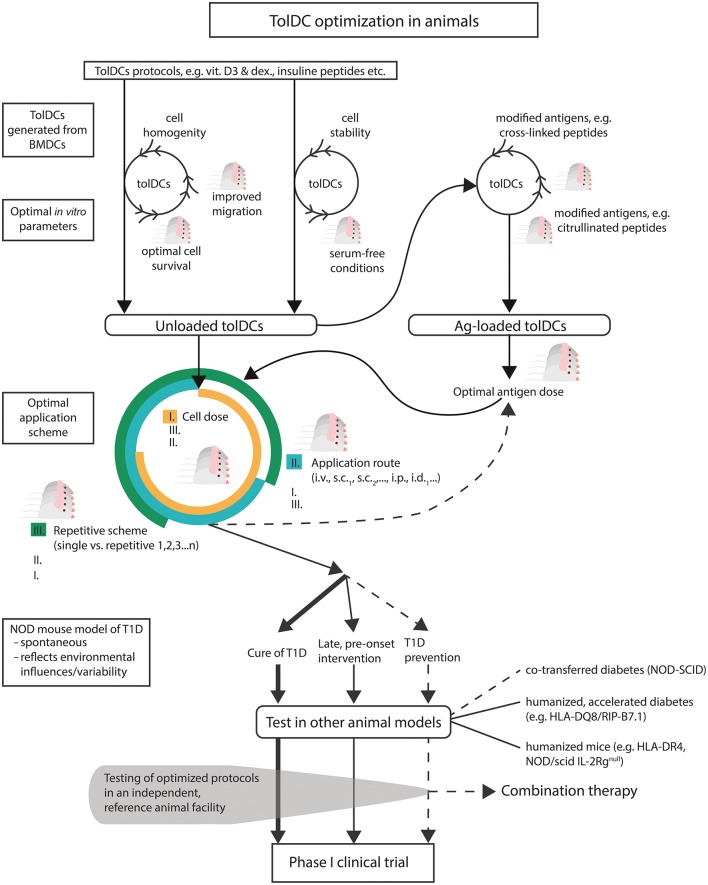
A scheme of suggested preclinical optimizations of tolDCs in T1D. Existing protocols of tolDCs should be optimized first *in vitro* for parameters such as cell homogeneity, serum-free conditions (to mimic closer human tolDCs protocols), enhanced stability, lengths of survival *in vivo*, and improved mucosal migration. The same parameters together with improved antigen modifications should be tested for antigen-loaded tolDCs, including optimal antigen doses. Next, combination of optimal tolDCs dose, regimen and application route should be determined. Effective and fine-tuned tolDC protocols should be tested in the spontaneous (NOD mouse) and also humanized models of T1D for not only prevention but also for their effect at the late prediabetic age or for the cure of diabetes. Finally, when possible, independent testing in a reference animal facility would be desirable before undertaking difficult translation from *in vitro* and mice to humans.

Another, more technical aspect of BMDC cultures for tolDCs, is the cell adherence and the fact that non-adherent and/or loosely adherent cells are harvested. This gray zone is further augmented by the tolerogenic protocols used. Various tolerogenic protocols or their modifications, such as the lengths of dexamethasone and vitamin D3 or D2 exposure, influence both *in vitro* adherence/yield of the tolDCs, but also the level of expression of their characteristic surface markers e.g., CD11c, CD40, CD80, CD86 (80, 81). Similarly, when reporting surface characteristics of *in vitro* generated tolDCs by flow cytometry in relation to diabetes prevention, modest or no SSC-A *vs*. FSC-A pre-gating should be used to document heterogeneity of cells that are actually injected to animals. Careful gating strategies have been documented, such as in the comparative study of clinical grade human tolDCs (80). Thus, mouse tolDCs protocols, especially those showing promising results in disease prevention, should be tested for cell homogeneity (Figure 1). This would also allow better comparison of their efficacies. Interestingly, preparation of mouse tolDCs from PBMCs was reported already back in 2000 (82). Although more laborious and demanding, perhaps the promising protocols should be re-tested with PBMC-derived mouse tolDCs for easier translation to clinical trials. No such attempt seems to have been published.

Some of these above described issues are indeed not present in human tolDCs that are prepared from PBMCs, and where closed culture systems/bags are preferred; their different materials were extensively tested and current best practice methods for preparation of immature or mature DCs do not rely on cell adherence to surfaces (83).

### Tolerogenic Protocols in T1D

An overview of tolerogenic protocols for *in vitro* generation of tolDCs in T1D is provided in Table 1. While some of the protocols were tested in several modifications and experimental set ups, others were reported only as a standalone study. Anti-sense oligonucleotides targeting expression of co-stimulatory molecules CD40, CD80, and CD86 led to induction of tolDCs with immature phenotype. Single *i.p*. administration of 2 × 10^6^ cells delayed diabetes onset and led to induction of splenic Tregs in NOD mice (36). Weekly injections from age of 8 to 12 weeks then completely prevented diabetes onset in NOD mice, possibly by enhanced expression of IL-7 as a survival factor for Tregs (84). The anti-sense oligonucleotides were tested in a phase 1 clinical trial (32, 33). In another study by this group, a microsphere delivery system of the anti-sense oligonucleotides was able not only to prevent diabetes but repeated (twice weekly) *s.c*. administrations also reversed hyperglycemia in new onset diabetic NOD mice (85). The second opened phase I clinical trial (34) is based on a protocol of antigen (peptide)-loaded tolDCs prepared in the presence of vitamin D3 with final lipopolysaccharide (LPS) activation (46). We have used a tolDCs protocol based on vitamin D2 and dexamethasone for diabetes prevention in NOD-SCID and NOD models of T1D, both with antigen-loaded and unloaded tolDCs prepared in FBS-supplemented or serum-free conditions, and with final tolDC stabilization by MPLA (42, 86). Using human DCs, we documented that the tolerogenic effect of such dendritic cells is controlled by p38, MAPK, ERK1/2 mTOR, and STAT3 signaling pathways (87). Comparable tolerogenic properties of vitamin D2 and vitamin D3 on *in vitro* cultured tolDCs were documented (88). Ferreira et al. showed that in NOD mice, vitamin D3 treated tolDCs migrate to PLNs and suppress T cell proliferation *in vivo* (61). The effect of vitamin D3 and dexamethasone on DCs was assessed at the transcriptome level and 11 genes that confer increased risk for T1D were found differentially expressed in tolDCs (89). It has been documented that vitamin D3 controls tolerogenic properties of DCs via a glucose metabolic pathway (61, 90). Morel‘s group showed that immature tolDCs were effective in disease prevention and associated with a Th2 cytokine shift (37, 60). Interestingly, IL-4 transduced tolDCs were reported by several studies to prevent diabetes in NOD mice, including at later pre-onset age (40, 54, 91) or even restore normoglycaemia in diabetic animals (53).

Several other protocols of tolDCs were reported in disease prevention, however they often included immature or semi-mature tolDCs without final stabilization (Table 1). Thus, immature DCs pulsed with ignored GAD65 antigen determinant GAD65_78−97_ (45), conditioning with IL-10 (43, 52), IL-25 (92), fungal extracts (93), *Lactobacillus casei* (67), carbon monoxide (94), or anti-CTLA4 Ab (95) were reported to prevent diabetes in animal models whereas DCs treated with PEGylated TLR7 ligand reduced diabetes and insulitis (38). Similarly, DCs pulsed with apoptotic bodies from the β cell line NIT-1 prevented diabetes in transgenic RIP-IFN-β NOD mice (44). Li et al. reported genetically modified DCs expressing T-cell co-inhibitory receptor BTLA that induced CD8^+^ T-cell tolerance and decreased diabetes in NOD mice (62). Thus, many promising protocols of tolDCs in T1D have been published, but often not further developed and/or optimized.

### Serum-Free, FBS, and Autologous Serum Conditions

Most of the published protocols of mouse tolDCs in T1D use fetal bovine serum (FBS) in the cell culture media (Table 1). However, Hasse et al. showed that DCs prepared in the presence of heterologous FBS and also pulsed with FBS on day 8, induced a Th2 cytokine shift in CD4^+^ T helper cells and increased Th2 cytokine production to FBS epitopes, including BSA, whereas tolDCs prepared in the presence of autologous mouse serum regulated immune responses in an antigen-specific manner. While unloaded tolDCs prepared in autologous mouse serum were ineffective in diabetes prevention in the induced RIP-LCMV mouse model, peptide-loaded tolDCs displayed some level of prevention. On the other hand, much better disease prevention was observed with tolDCs (irrespective of a peptide-loading) prepared in the presence of heterologous FBS (52). Later they reported similar results in NOD mice; antigen-loaded tolDCs were effective in disease prevention when cultured in autologous serum and this effect was accompanied with an increase of Foxp3^+^ Tregs in peri-insulitic infiltrate (43). These data, together with data by Feili-Hariri et al. who reported no additional beneficial effect of antigen-loading for tolDCs prepared in the presence of FBS, point to the possible role of FBS in immune mechanisms (Th2 *vs*. Tregs) by which tolDCs may operate (37). Nevertheless, human cell therapies are manufactured in serum-free conditions. Tolerogenic DCs cultured in serum-free conditions were shown to display superior characteristics compared to FBS-prepared ones as regards their tolerogenic phenotype, induction of Tregs in PLNs and also disease prevention in already prediabetic 8–9 weeks old NOD mice (66). While we observed almost no effect on phenotypic differences in vitamin D2 and dexamethasone generated tolDCs, both unloaded and antigen-loaded tolDCs displayed a tendency to better disease protection when prepared in serum-free conditions (42). Interestingly, while tolDCs propagated by GM-CSF alone displayed similar properties in serum-free *vs*. FBS-supplemented media, serum-free conditions were superior for GM-CSF+IL-4 generated tolDCs (73). Mouse DCs generated as an antitumor vaccine also possessed better phenotypic and functional characteristics when generated in serum-free conditions (96). Based on this evidence as well as the fact that it would bring animal experiments one step closer to clinical testing, mouse protocols of diabetes-preventive tolDCs should be tested and optimized in serum-free conditions (Figure 1). However, only a few studies included or compared serum-free culture conditions (Table 1). Testing of promising protocols in serum-free media is not difficult and in our opinion is necessary in preclinical studies.

### TolDCs Stability

Among many parameters of tolDCs, their stability is of upmost importance (Figure 1). DCs are sufficient for CD8^+^ T-cell priming *in vivo* (97) and in pathogenesis of T1D they are instrumental for mounting effector T-cell responses involved in β-cell destruction (98, 99). Tolerogenic DCs used as a therapy for autoimmune diseases or allergies are likely to encounter inflamed environments and their compromised stability could lead to a change toward immunostimulation with possible dangerous consequences, especially in case of antigen-loaded tolDCs. Similarly, hyperglycemia and consequent oxidative stress may alter tolDCs effectivness by reducing their T regulatory capacity (86). Naranjo-Gómes et al. compared the stability of clinical grade human tolDCs stabilized by a cytokine mix and showed that vitamin D3, rapamycin or dexamethasone conditioning suppressed allogeneic proliferations and IFN-γ production and led to stable tolerogenic phenotype *in vitro* (80). Similarly, dexamethasone-treated tolDCs further stabilized by monophosphoryl lipid A (MPLA) displayed a stable and enhanced migratory phenotype (100). We have reported stable mouse tolDCs prepared with vitamin D2/dexamethasone and exposed for 24 h to MPLA (42). Other protocols used LPS (36, 46) or its combination with IL-10 (69) or IFN-γ (81). Both protocols that progressed to clinical trials used stabilized tolDCs in animal experiments (36, 46).

There are also reports of unstable tolDCs not suitable for cell therapies (101). Attempts to further harness stability of tolDCs (Figure 1) were undertaken e.g., DCs transduced with human 25-hydroxyvitamin D 1α hydroxylase (102), whereas Chai et al. reported that recombinant OCILRP2-Fc (Osteoclast inhibitory Lectin-related Protein 2) inhibits LPS-driven maturation and differentiation of BMDCs (103). The modulating effects of vitamin D3, rapamycin dexamethasone, TGF-β and IL-10 were also assessed in a comprehensive study with clinical grade tolDCs by Boks et al. who nicely documented superior tolerogenic effect of IL-10, including stability of DCs and induction of Tregs with strongest suppressor activity on T cells (104). Thus, propagation of tolDCs with IL-10 (39, 43, 52) might be superior to GM-CSF+IL-4 only. Unfortunately, many of the animal protocols use immature DCs without terminal differentiation and/or their stability was not adequately addressed (Table 1). Since tolDCs stability is critically important for translation to humans, it should be thoroughly addressed for any protocol developed with this intention.

### Optimal tolDCs Dose, Application Schemes

While many different tolDCs protocols were tested in animal models of T1D for their diabetes preventive or even curative effect, experiments optimizing such protocols are either missing or not published. Only a few studies actually reported testing multiple doses of tolDCs and/or single *vs*. multiple repetitive schemes or their combinations (Table 1). Cell doses varied from 2 × 10^5^ to 3 × 10^6^ tolDCs. More than one application scheme have also been reported (39, 54, 60, 63, 65) (Table 1). Although human clinical trails are usually only inspired by doses from animal testing and more data are available from the use of DCs in cancer immunotherapy, more optimizations of the promising tolDCs protocols in T1D should be carried out in preclinical testing (Figure 1). Such optimization is perhaps not scientifically very appealing and may be difficult to get published, yet such important animal data are lacking.

## Route of Administration and tolDCs Migration

Several application routes including *i.p., i.v., s.c*., and also *i.d*. were used in animal studies of tolDCs in T1D (Table 1). Many animal studies of diabetes prevention used *i.p*. (36, 39, 42–44, 52, 61, 87) or *i.v*. (37–40, 53, 54, 60, 66, 69) routes of administration. Preferred migration to PLN compared to mesenteric lymph node (MLN), spleen and inguinal lymph node (ILN) (29, 42), and increased accumulation of bone marrow-derived DCs in pancreas and liver (61) was documented after *i.p*. administration. Intravenous application also targeted PLNs but together with the spleen (29, 37, 54). Creusot et al. provided a very elegant study comparing *in vivo* homing of bone marrow-derived DCs after *i.v*. and *i.p*. administrations. While *i.p*. administration led to accumulation of DCs preferentially in PLNs and also omentum, the *i.v*. route targeted spleen as well as PLNs and lung-draining LNs but very few cells were detected in MLNs, ILNs, and LNNs (29). Intraperitoneal and intravenous application routes are also being used in current human trails with tolDCs [reviewed (31)]. The two tolDCs clinical trials in T1D (32, 34) are using subcutaneous and intradermal application routes, respectively. While the *i.d*. application was reported in a proof-of-concept animal study without diabetes incidence testing (46), *s.c*. applications at locations such as the abdominal flank overlying the pancreas (63, 64) the footpad (45, 65) or unspecified (62) were referred for tolDCs in T1D. Tolerogenic DCs were nicely documented in the subcutaneous compartment after *s.c*. injection and their accumulation in PLNs was reported (64). The *s.c*. abdominal application close to the pancreas projection was described as preferable for accumulation in PLNs [reviewed (30)]. Most of the animal studies in T1D included *i.p*. and *i.v*. applications of tolDCs. A comparison of all currently used application routes carried out with the same tolDCs protocol is however missing (Figure 1). TolDCs were reported to survive about 1–2 weeks *in vivo* (29). We detected live tolDCs *in vivo* for up to 12 days following *i.p*. administration (42). Nevertheless, more experiments on the lengths of *in vivo* survival of tolDCs are needed, as application routes may also influence survival of tolDCs (Figure 1).

Increased migration of tolDCs to PLNs, pancreas and/or other mucosal LNs is a highly desirable feature of therapeutic tolDCs in T1D, so that they have a better access to T1D-related antigens. Priming of diabetogenic T cells in NOD mice occurs in PLNs and gut-associated LNs (105). The importance of PLNs in T1D may be also supported by a study showing that surgical removal of PLNs at 3 weeks (but not 10 weeks) prevents development of diabetes in NOD mice (106). Among molecules that may improve migration and mucosal homing of tolDCs, CCR7 expression was documented as critically important [reviewed (28, 107)] while L-selectin may be engaged for entering the LNs (108). Migration pattern is an important parameter for tolDCs. While tolerogenic agents (e.g., dexamethasone, vitamin D3) may decrease CCR7 expression [reviewed (109)], MPLA activation and terminal differentiation of tolDCs increases CCR7 and CXCR4 expressions and thus improves their migratory capacity (100). Similarly, rapamycin was reported to increase CCR7 expression in human DCs (110). Final stabilization of clinical grade human tolDCs by exposure to TNF-α, IL-1β, and PGE (2) also increased their migration efficacies (104). On the other hand while IL-10 was reported to improve tolDC stability and Treg-mediated tolerogenic capacity, it also impairs mucosal migration of DCs by downregulation of their CCR7 expression (111). Interestingly short but not continuous exposure to IL-4 toward the end of GM-CSF propagation of DCs was reported to enhance their trafficking efficacy (76). Retinoic acid, but not expression of CD103 by DCs, was shown to be critical for mucosal α4β7-mediated homing of T cells (112). Protocols of tolDCs in T1D should be thus optimized not only for their stability and tolerogenic capacity but also migratory efficacy to secondary lymphoid organs. However this is rarely the case (Table 1).

## Antigen-Unspecific *vs*. Antigen-Specific Approach, Antigen Dose

Several studies reported diabetes prevention by unloaded tolDCs (Table 1). Feili-Hariri et al. reported tolDCs generated without an antigen, to prevent diabetes after *i.v*. administration to NOD mice (37). Pulsing tolDCs with a mixture of hsp60 and two GAD65 peptides did not augment their ability to prevent diabetes development. Diabetes prevention was possibly mediated by an induced Th2 shift in treated animals (60). Later, they also reported tolDCs transduced for IL-4 expression to prevent diabetes in NOD mice when applied *i.v*. at the age of 5 weeks but also at 7–8 and 10 weeks, i.e., in animals just before the onset of diabetes and with progressed insulitis (54). In both studies, migration of tolDCs to spleen and PLNs was nicely documented. Another approach was documented in the study by Ma et al. who also used antigen-unloaded tolDCs that were treated *in vitro* with NF-κB-specific oligodeoxyribonucleotide (ODN) for diabetes prevention in older 6–7-week-old NOD mice (41). Interestingly, when these tolDCs were used in an antigen-specific manner i.e. pulsed *in vitro* with islet lysate, the diabetes preventive effect was lost. Unloaded tolDCs treated with antisense oligonucleotides against costimulatory molecules (CD40, CD80, and CD86) delayed diabetes in NOD mice, but not if pulsed *in vitro* with cell lysate from the NIT-1 β-cell line. The diabetes prevention was associated with increased numbers of CD4^+^CD25^+^ T cells (36). Creusot et al. then published a study using IL-4 transduced tolDCs. When applied *i.v*. to 12-week-old NOD mice, these antigen-unspecific tolDCs migrated to spleen and PLNs and were able to significantly delay or prevent onset of diabetes in pre-diabetic animals (40). Tolerogenic DCs propagated with GM-CSF+IL-4 from BMDCs isolated from GM-CSF-treated NOD mice also decreased development of diabetes when applied to 3-week-old NOD recipient mice (69). An elegant study by Tai et al. in which multiple parameters were assessed, showed that tolDCs propagated with GM-CSF+IL-10 suppressed diabetes and insulitis in two animal models, the NOD and HLA-DQ8/RIP-B7.1 mice (39). While *in vivo* stimulation of DCs with PEGylated TLR7 ligand delayed diabetes and reduced insulitis upon transfer to prediabetic NOD mice, when these DCs were pulsed with GAD65_515−524_ peptide they significantly increased insulitis compared to both controls but also unloaded PEGylated TLR7 ligand–treated DCs (38). Vitamin D2 and dexamethasone conditioned tolDCs also prevented diabetes in NOD-SCID and NOD models, but this effect was lost if tolDCs were loaded with mouse GAD65, its immunodominant peptide no. 35 or even with a control protein - OVA (42, 86). Remarkably, multiple *s.c*. (abdominal flank) injections of immature DCs treated with antisense oligonucleotides against costimulatory molecules restored normoglycaemia in already diabetic NOD mice (63).

This review is not listing all tolDCs studies in animal models of T1D, but the above described examples well-document that unloaded tolDCs, often without stabilization, or immature DCs were effective in disease prevention, in stopping clinical onset of diabetes at 12 weeks of age or even restoring normoglycaemia in already diabetic NOD mice. The last two stages may correspond to individuals that could be diagnosed as having high risk of progression to T1D or new onset T1D patients. On the other hand, modifications with antigen rendered these protocols ineffective or even worsened insulitis (36–38, 41, 42). This scenario is surprisingly different from the expectations with antigen-loaded tolDCs that are being developed aiming for a more specific and efficient tolDCs therapy in T1D.

Among studies dealing with an antigen-specific approach, Marin-Gallen et al. showed that immature DCs loaded with apoptotic bodies from the NIT-1 β-cell line, but not from control SV-T2 embryonic cell line, prevented diabetes. Unloaded control immatured DCs (iDCs) had no preventive effect as well (44). The importance of using autologous serum and not heterologous antigens (i.e., FBS serum) for antigen-loaded (insulin B9-23 and B15-23 peptides) tolDCs was clearly documented both by diabetes prevention and Tregs induction (43). Nevertheless, when splenocytes from disease-protected animals using insulin peptide-loaded tolDCs were retested for their regulatory potential in the adoptive NOD-SCID co-transfer model, they caused more rapid and a 100% onset of diabetes compared to controls (43). Later Looney et al. investigated the effect of serum-free *vs*. FBS-supplemented culture condition on tolDCs loaded with GAD65217-236 peptide. They demonstrated that only tolDCs cultured in serum-free medium prevented diabetes in NOD mice, induced Tregs and lasting β-cell specific T-cell responses (66). Recently, Lo et al. showed that immature DCs cultured in the presence of FBS and pulsed with subdominant or ignored peptide determinants, but not with immunodominant insulin peptide B9-23, decreased diabetes incidence in already 9-week-old NOD mice (65). Thus, this is another example of antigen-specific iDCs being less effective in disease prevention.

At present, unloaded tolDCs seem to represent more suitable choice for clinical testing, both from the point of their efficacy as well as safety. More research is needed in the field of antigen-loaded tolDCs, as delivery of immunodominant epitopes may pose an increased risk of disease acceleration. Such protocols should be well-optimized in animal models of T1D (Figure 1). One risk factor may represent the after death fate of antigen loaded tolDCs. Antigens from therapeutic tolDCs may cause sensitization via processing and presentation by recipient APCs (113). Another parameter to consider is the antigen dose (Figure 1). An elegant study by Smyth et al. documented that low doses of antigen presented by both immature and mature DCs, but also unloaded mature DCs, induced weak TCR signaling via Akt/mTOR pathway and expansion of Foxp3^+^ Tregs. On the other hand, high antigen doses led to strong Akt/mTOR signaling and expansion of Foxp3^−^ Th cells. This effect was modulated by T-cell-produced IL-6. The DC phenotype was thus less important than antigen dose (114). This finding corresponds with data from other immunointervention strategies such as mucosal delivery of autoantigens in which lower autoantigen doses were often associated with more satisfactory results (56, 115, 116).

A new perspective for antigen-loaded tolDCs in T1D is perhaps represented by post-translationally modified T-cell epitopes. Increasing evidence suggests that post-translationally modified epitopes may play a role in autoimmune diseases including T1D, especially during the not so well understood initial phases of autoimmune responses (117). Enzyme modifications such as citrullination by peptidyl deiminases or deamidation by tissue transglutaminases, as well as cross-linked peptides or aberrant mRNA translation, were described as sources of neo-epitopes relevant in T1D (118–120). The neo-epitopes may explain how T-cell tolerance (T-cell deletion and peripheral tolerance) is circumvented in autoimmunity and also represents an interesting link to an initial environmental insult such as stress or viral infection that triggers their increased genesis (117), which has been implicated in pathogenesis of T1D. In type 1 diabetes cross-linked peptides of proinsulin to other β-cell peptides (HIPs) were reported to be recognized by pathogenic CD4^+^ T cells (121). Autoreactive CD4^+^ T cells have been implicated in the initial breakdown of tolerance by providing help to autoreactive CD8^+^ T a B cells. In a rheumatoid arthritis human phase I trial with four cintrullinated peptides, there was documented a reduction in effector T cells in 11 out of 15 patients and, to a lesser extent, also an increase of Tregs (122). Thus, T1D-related neo-epitopes represent interesting and promising antigens to be tested in antigen-specific tolDCs therapies for T1D (Figure 1).

## Prevention *vs*. Treatment

Several tolDCs protocols prevented diabetes in animal models (mostly NOD mouse) of T1D diabetes (36–39, 42, 44, 66, 69) (Table 1). There are however a few tolDCs protocols that prevented diabetes in older NOD mice with advanced insulitis or at the age just before usual clinical onset of diabetes. Thus, Feili-Hariri et al. reported prevention of diabetes in 10-week-old NOD mice, Lo et al. showed in 2 papers diabetes prevention in NOD mice that were treated from 9 weeks of age (45, 65), and Creusot et al. prevented diabetes in already 12-week-old NOD mice using immature DCs transduced to express IL-4 (40). A few tolDCs protocols have been shown to cure diabetes/restore normoglycaemia in already diabetic NOD mice. Single *i.v*. administration of DCs electroporated with IL-4 mRNA reversed hyperglycemia in diabetic NOD mice to fluctuating levels for up to 300 days and prevented diabetes in 12-week-old prediabetic animals (53). Later Di Caro et al. restored normoglycaemia in diabetic NOD mice by eight *s.c*. injections of immature DCs treated with antisense oligonucleotides against costimulatory molecules (63). The same group then showed reversal of hyperglycemia with antisense oligonucleotides, and also in combination with insulin B9-23 peptide, for at least 24 weeks (64). Indeed, these are the good candidate protocols for translation to clinical testing (Table 1; Figure 1).

Diabetes preventive protocols should be further optimized and also tested as early pre-onset interventions or for diabetes reversal. In addition they should be also tested in combination therapies (Figure 1). There are however very limited published data on tolDCs protocols that tested, but did not prevent, diabetes in the late pre-onset age, or failed to cure already diabetic animals. One such published study is by Pujol-Autonell et al. who showed DCs loaded with apoptotic bodies from the NIT-1 β-cell line did not reverse diabetes in NOD mice (68). In addition, this study also probed a combination approach with rapamycin and reported a negative outcome. Nevertheless, tolDCs were propagated in GM-CSF alone, and thus optimized variants of this protocol may still have a different outcome. More attempts of combination therapy, especially when using well-optimized diabetes preventive tolDCs, should be undertaken (Figure 1). There are examples of combination therapies tested in closely related applications e.g., prevention of T1D by acetylated dextran microparticles with rapamycin and pancreatic peptide P31 (123).

## Animal Models

The most common animal model in T1D research is the NOD mouse. It displays several important similarities, but also some differences, compared to human T1D. While multiple manipulations have been reported to prevent disease in NOD mice (2), this goal has not yet been achieved in humans. The main advantage of this model is that unlike in many other autoimmune diseases, it spontaneously develops the disease with incomplete penetrance, thus reflecting well the contribution of environmental factors in T1D. Similar to human T1D, NOD mice possess polygenic genetic susceptibility with prevalence of MHC genes. Furthermore, diabetes onset is preceded by an increased number of circulating autoreactive T cells and autoantigens, including the most important ones to (pro) insulin, GAD65, IA-2, and others. In the NOD mouse, the initiating antigen seems to be (pro) insulin, whereas in human T1D more antigens can give rise to autoimmune reactivity [reviewed (124)]. Although the cellular composition of pancreas infiltrating cells is also similar, the histological characters of insulitis differ, being more severe and frequent than in human T1D (48). NOD mice also have the advantage of less severe ketoacidosis and thus relatively long survival after diabetes onset, allowing easier set-up of experiments involving insulin treatment and reversal of diabetes. NOD mice also have the advantage of less severe ketoacidosis and thus relatively long survival after diabetes onset, allowing easier set-up of experiments involving insulin treatment and reversal of diabetes. Thus, the NOD mouse has been established as the most frequently used proof-of-concept animal model in T1D.

There are however also differences and weaknesses of the NOD mouse model specifically applying to DC therapies. Several studies reported abnormalities in the development of myeloid cells in NOD mice (125, 126), including defective maturation of myeloid DCs via IDD10/17/18 (50), while a later gene profiling study revealed over 300 differences in NOD DCs upon LPS stimulation, including expression from a cluster of 16 INF- α/β target genes (127). Apart from the defect in the maturation of NOD DCs (128), a lower responsiveness of bone-marrow progenitors to GM-CSF propagation was also described (72). Other studies documented that BM-derived DCs from NOD mice possess a hyperinflammatory profile with elevated NF- κB levels, increased IL-12 production and reduced ability to induce proliferation of the Treg population (129). A lower stimulatory T-cell capacity and a defect in CD8^−^ dendritic cells have been reported more recently (130). These characteristics may indeed negatively influence cell yields as well as sensitivity for tolDC protocols tested. Nevertheless, despite the above mentioned DC defects, various tolDC protocols tested in NOD mice have yielded fully functional tolerogenic DCs with capacity to prevent T1D (36, 42, 61) or even reverse hyperglycaemia in recent onset diabetic recipients (53, 63, 64), indicating that NOD mice represent a satisfactory model for preclinical testing of tolDCs.

The other spontaneous rodent model, the BB rat, displays a defect in thymic epithelial cells and severe lymphopeniea as well as altered maturation of DCs (1, 131, 132) and no preclinical testing of tolDCs seems to have been reported in this model. Very recently, a tolDCs and cDCs comparison was carried out in autoimmune-prone and resistant rats, but not in BB rats (133).

The NOD-SCID mouse model is used for adoptive co-transfer of diabetes (51). While the observation period for diabetes incidence is much shorter than in NOD mice, a titration of diabetogenic splenocytes and their capacity to transfer diabetes across different experiments should be controlled to ensure similar sensitivity of the model. In addition, because of the relatively small number of T cells, homeostatic expansion of T cells may also influence this model. Nevertheless, comparable data in tolDCs therapies were obtained using both models (39, 42). Other models of accelerated diabetes e.g., LCMV-RIP and NOD RIP-IFN-β have also been used in tolDC-based therapies of T1D (44, 52). The induced animal models represent a more challenging scenario for diabetes prevention or treatment and should therefore be included in preclinical optimization of tolDCs.

In addition, humanized mouse models were employed to bring testing closer to clinical trials and to assess immune responses in the context of risk human HLA molecules such as HLA-DQ8/RIP-B7.1, or HLA-DR4 transgenic mice (39, 46). The NOD/scid IL-2Rg^null^ humanized mouse developed as a preclinical model for rapid *in vivo* evaluation of human DCs-based therapies, including *ex vivo* T-cell responses with recovered human T cells (134). The humanized mouse models bring preclinical optimization of tolDCs one step closer to translation to clinical trials (Figure 1).

## Concluding Remarks

The development of DC-based therapies consists of multiple steps and involves many parameters. While it is difficult to optimize all of them given their interplay, we think it is important to assess single parameters side by side, not only *in vitro* as it has been done for example with some protocols and clinical grade tolDCs, but also in animal models of T1D. Published animal studies on tolDCs in T1D do not address this issue sufficiently. Since tolDCs therapies will probably evolve incrementally, optimization of various parameters and better understanding how they influence efficacies of tolDCs *in vivo* in animal models is important.

In this review we have assessed tolDCs protocols reported in animal models of T1D for parameters such as culture conditions comprising tolDCs propagation, homogeneity, serum *vs*. serum free conditions, and stability or terminal differentiation. Next we have discussed how cell dose, single *vs*. repetitive application schemes, routes of administration including migration properties of tolDCs, unspecific *vs*. antigen–specific approach were researched and optimized in animal, mainly NOD, models of T1D. Effective and fine-tuned protocols should be then tested and reported not only for prevention but also as an intervention at the age of advanced pre-diabetes or cure of T1D in multiple animal models including humanized mice. Modifications of autoantigens and combinatorial approaches were briefly mentioned.

After decades of research to find a cure or effective secondary prevention for type 1 diabetics, DC-therapies represent a relatively new approach with remarkable achievements. A translation to humans seems optimistic as a few tolDCs protocols even reversed diabetes in NOD mice. This most frequently used spontaneous model of T1D is sometimes criticized for the easiness to prevent diabetes. Nevertheless, this is only easy at age of 3–4 weeks or even prenatally and thus no comparative human data exists. Another lesson from animal models is that among the main two approaches of using unloaded or antigen-loaded tolDCs, more data are at present available for an antigen-unspecific approach, yet this may change in the near future. As discussed in several subchapters of this review, almost all parameters of tolDCs would benefit form a more thorough optimization for translation to a clinical testing, starting from *in vitro* parameters, such as serum-free conditions (42, 52, 66) and stability testing, to optimal application scheme (e.g., multiple doses were used for reversal of diabetes by tolDCs (63), to the use of various mouse models in preclinical experiments. There are also some unexpected factors such as the increased effectiveness of lower tolDCs doses (114). In addition, some other parameters not yet tested in animals could be important in patients e.g., the effect of glycaemia control on functional properties of patient-prepared tolDCs [reviewed (86, 135)].

Although many tolDCs protocols in T1D were reported, we think they should be thoroughly optimized in animal models as tolDCs therapies in patients comprise not only safety issues, but also involve significant time, costs and a great deal of the patient's hopes.

## Author Contributions

DF wrote the first draft of the manuscript. DF, LP-J, JG, ZK, AF, TH, and RŠ contributed in design, scientific insights, manuscript writing, editing and proof reading.

### Conflict of Interest Statement

LP-J and RŠ are named inventors in a related patent “TolerogenicDendritic Cells, Methods of Producing the Same, and Uses Thereof” PCT/EP2015/074536 which describes methods for the preparation of stable semi-mature tolerogenic DC. LP-J and RŠ were employed by company SOTIO a.s. The remaining authors declare that the research was conducted in the absence of any commercial or financial relationships that could be construed as a potential conflict of interest.
